# Cross-sector cooperation in health-enhancing physical activity policymaking: more potential than achievements?

**DOI:** 10.1186/s12961-016-0103-6

**Published:** 2016-04-29

**Authors:** Riitta-Maija Hämäläinen, Arja R. Aro, Cathrine Juel Lau, Diana Rus, Liliana Cori, Ahmed M. Syed, the REsearch into POlicy to enhance Physical Activity (REPOPA) Consortium

**Affiliations:** National Institute for Health and Welfare, Mannerheimintie 166, 00271 Helsinki, Finland; Department of Public Health, Unit for Health Promotion Research, University of Southern Denmark, Niels Bohrs Vej 9, 6700 Esbjerg, Denmark; Research Centre for Prevention and Health, The Capital Region of Denmark, Ndr. Ringvej 57, Afsnit 84/85, 2600 Glostrup, Denmark; Administrative and Communication Sciences, Cluj School of Public Health, College of Political, Babes-Bolyai University, Cluj-Napoca, Romania; Institute of Clinical Physiology, National Research Council, Via Moruzzi 1, Pisa, 56124 Italy; NHS England, 80 London Road, London, SE1 6LH United Kingdom

## Abstract

**Background:**

The cooperation of actors across policy fields and the need for cross-sector cooperation as well as recommendations on how to implement cross-sector cooperation have been addressed in many national and international policies that seek to solve complex issues within societies. For such a purpose, the relevant governance structure between policy sectors is cross-sector cooperation. Therefore, cross-sector cooperation and its structures need to be better understood for improved implementation. This article reports on the governance structures and processes of cross-sector cooperation in health-enhancing physical activity (HEPA) policies in six European Union (EU) member states.

**Methods:**

Qualitative content analysis of HEPA policies and semi-structured interviews with key policymakers in six European countries.

**Results:**

Cross-sector cooperation varied between EU member states within HEPA policies. The main issues of the cross-sector policy process can be divided into stakeholder involvement, governance structures and coordination structures and processes. Stakeholder involvement included citizen hearings and gatherings of stakeholders from various non-governmental organisations and citizen groups. Governance structures with policy and political discussions included committees, working groups and consultations for HEPA policymaking. Coordination structures and processes included administrative processes with various stakeholders, such as ministerial departments, research institutes and private actors for HEPA policymaking. Successful cross-sector cooperation required joint planning and evaluation, financial frameworks, mandates based on laws or agreed methods of work, communication lines, and valued processes of cross-sector cooperation.

**Conclusions:**

Cross-sector cooperation required participation with the co-production of goals and sharing of resources between stakeholders, which could, for example, provide mechanisms for collaborative decision-making through citizen hearing. Clearly stated responsibilities, goals, communication, learning and adaptation for cross-sector cooperation improve success. Specific resources allocated for cross-sector cooperation could enhance the empowerment of stakeholders, management of processes and outcomes of cross-sector cooperation.

## Background

The concept of health-enhancing physical activity (HEPA) has been defined as any form of physical activity that improves health and has the fewest possible undesirable side effects. HEPA is characterized by intensity, duration and frequency [1, 2]. The complexity of low physical activity, sedentarism or inactivity of people is due to to multiple causes of the problems, their cross-sectoral nature, and a lack of clarity of solutions and changing behaviours in people. In tackling such complex issues with a view to improving health, efficient governing between sectors is needed [3, 4]. However, to date, only a small number of analytical papers address physical activity policies [5–11] and none address the cross-sector cooperation for HEPA to achieve better health.

Complex problems need complex adoptive systems and health professionals need to adopt a whole-systems approach so as to tackle multiple problems and behaviours simultaneously [12]. One of the possible solutions is management of complex issues by better governance [13, 14]. Governance has been defined as a process of continuing interaction between participants inside and outside the formal structures of government [15]. The processes require a diversity of frameworks and applications in order to gain attention and evaluation. However, the ‘siloization’ or ‘pillarization’ of the public sector for certain issues has hampered collaboration between administrative entities and sectors [16]. Nevertheless, various concepts of collaboration with slightly different meanings have been used in different situations with their own implications. Such common, synonymously used concepts are coordination, coherence, collaboration, integration and inter-sector initiative [17].

In this article, cross-sector cooperation is used to describe the joining up of different sectors to work together so as to produce a broader view, so that ministries, agencies, and local service centres can make a better contribution to cross-sector cooperation. Thus, cross-sector cooperation can reduce conflicts between different policies and tackle wider issues by promoting policies that are better interconnected and mutually supportive. Cross-sector cooperation may promote innovation by finding new ways and means through a variety of experiences, skills, and backgrounds for services and by making better use of resources by removing overlaps [16]. In this article, the sector refers to different levels of decision-making (federal, regional, local; the vertical dimension), different governmental fields (such as health, sport, education, transport; the horizontal dimension of collaboration) or collaboration between public, private and third sectors. As we studied policies, we defined policy documents as written documents that contain strategies and priorities, define goals and objectives, and are issued by a part of the public administration. In view of the particular importance of national policy documents for health, the primary analysis focused on documents issued by national, regional or local bodies (adapted from [17, 18]).

The interest within public health policy for cross-sector cooperation has been around for a long time. In this article, cross-sector collaboration is understood and used as it is in the public health perspective. Initially, intersectoral actions meant coordinated and direct actions to improve population health; however, in practise the term was used more often to underline collaboration between public and private sectors. However, since 1978, the idea of cross-sector cooperation has been used more in the spirit of partnership and coordination with other sectors in public health [19–24].

According to several studies, coordinated actions at multiple levels promoting health are usually more effective than single interventions [25, 26]. The Canadian Public Health Agency and WHO [27] stated that multiple governance actions may be more effective than single ones. However, the integration of HEPA into policies of other sectors seems to be more sporadic rather than intentional and well thought-out approaches. Evidence from other sectors, such as environment and public–private–non-governmental partnerships, indicates that partnerships between the public and private sectors and non-governmental organisations often lack commitment from top management, the interests of the parties involved sometimes collide, and the implementation can be burdensome and cause conflicts [28–31]. Moreover, despite the current popularity of the partnership discourse and its relation to cross-sector cooperation, little is known about the actual impacts of these endeavours.

The complex problems, which are marked by value divergence, knowledge gaps and uncertainties, are not tackled successfully through standardized approaches [32]. Highly complex issues require new modes of producing, mobilising and implementing knowledge through participatory and interdisciplinary approaches, which would have broker roles between researchers and stakeholders as well as academics and local people. Major coordination problems remain in both the vertical (between levels of administration) and horizontal (between different policy areas and sectors such as education, health and transport) dimensions. The implementation of cross-sector cooperation resides in various factors such as the compatibility of sectors, the scope of which sectors can address improvements in health-related determinants, and the costs that accrue from taking health into account in other sectors [33]. To enhance political legitimacy and accountability and to take HEPA into account in other policies, where partners are reluctant, the focus and emphasis needs to be more on the benefits and values of cross-sector approaches than actual HEPA policy [7, 34].

This article discusses and analyses the governance structures for implementation of cross-sector cooperation in HEPA policymaking in six European Union (EU) member states. Our specific research questions concerned the role of stakeholders in HEPA policymaking, how the HEPA policies address cross-sector cooperation and what kinds of mechanisms existed.

## Methods

Data reported in this article has been collected as part of REPOPA (REsearch into POlicy to enhance Physical Activity) [35] – a cross national project including EU six member states between 2012 and 2013. This particular paper presents case findings from all six countries: Denmark, Finland, Italy, the Netherlands, Romania and the United Kingdom. For the overall REPOPA project, it was important that the partners represent not only universities and research institutes producing research evidence, but also implementation, policy and practice organizations bringing in interests, values and priorities [35]. Furthermore, to understand cultural and other contextual challenges, REPOPA project partners come from different regions. The policy selections and topics of the policies have been described previously [35, 36]. Given the complex processes of policymaking, policy analysis of HEPA policy documents was conducted in order to gather the perspectives of the cross-sector cooperation in shaping HEPA policies. The study selected two to five policies within HEPA in the six countries as the basis for a content analysis of policies and policymaking processes. From the national, regional or local levels of HEPA policies, 21 policies were selected. Content analysis of policy documents and interviews were conducted.

The content analyses of the policy documents and the interviews complemented each other to identify cross-sector collaboration. The intention was to facilitate finding differences and similarities between countries in cross-sector cooperation and to take into consideration contextual differences through interviews.

### Content analysis of policy documents

Based on the literature review for the project proposal and its update upon acceptance of funding, the study was undertaken using a qualitative descriptive approach inspired by the political sciences [37, 38], public health sciences [39, 40] and the multidisciplinary field of knowledge transfer, knowledge utilisation and lesson learning [41–43].

The selection criteria for the policies were that they had to constitute the endpoint for completed policy development process and make a major HEPA initiative at local, regional or national level. The policies selected had been developed between 2001 and 2013, and were all being implemented, providing richness of the data and potential for comparison between policies and countries [35]. All policies were in their national language and were analysed by the REPOPA research team members.

The content analysis of HEPA policy documents followed the ideas of Ritchie and Spencer [44], and consisted of the process of analysing policy documents by issues and topics with the help of a set of guiding questions (e.g. what constituted cross-sector cooperation and what kinds of tools and mechanisms were used). After mapping the issues and topics, the HEPA documents were further analyzed so as to identify the stakeholders and structures of cross-sector cooperation for each policy. Each HEPA policy was reread, sifted, charted and sorted according to the key issues, topics and themes, confirming the patterns of cross-sector cooperation.

The REPOPA team prepared a common guideline for all partners for the content analysis of policy documents and what to look for among the selected policy documents in relation to cross-sector cooperation. Thereafter, the findings were reported in English and collated into one report. The implementation phase of the policies was not included in the analysis, since that lies outside the scope of this study.

The common guideline that was developed covered the selection of policies, theoretical models of the policymaking phases, the focus of analysis in relation to topics, goals and processes, identification of stakeholders, the process description of the policy analysis and instructions for the HEPA policy analysis of the cross-sector cooperation among others in policymaking, and a schematic example of the analysed text of a policy.

At the content analysis stage, the policy documents were reviewed by the REPOPA country teams to identify stakeholders, processes and sectors included in cross-sector cooperation. This also provided an opportunity to identify broader political forces (for example, stakeholder positions) influencing policy decisions and to define how cross-sector cooperation was arranged in the policymaking process.

### Semi-structured interviews, interview guide and topics covered

During the HEPA policy document analysis, key informants for each policy were identified for face-to-face semi-structured interviews in order to verify the findings of the content analysis stage and gather information on any gaps in the cross-sector cooperation. The purposeful sampling of 86 interviewees (on average three to five interviews per policy paper) in six countries was based on the selection criteria of the interviewees directly involved in the policymaking process and able to report on the cross-sector cooperation [36]. In each policy, one of the interviewees was from the organisation responsible for the policy. The interviewees had been involved in the policymaking processes of the policy about which they were interviewed. The interviewees were policymakers, researchers, public sector officials or other influential stakeholders. All the interviewees were contacted by email or phone by the research team in each country with basic information on the project and consent forms in the local language. The interviews were conducted in the local language by research team members with backgrounds in health and social sciences, recorded when accepted and transcribed for the analysis. When two interviewees refused recording, notes were taken and included into the study as a text of the interviews. An interview guide was developed by the REPOPA team. In the guide, the questions for the policymaking process were split into agenda-setting and policy development phases. A consent form, description of the research project and preliminary list of questions were also provided. The interviews were conducted according to the document analysis of the policies.

To adapt the interviews for each context, each country team conducted between one and three pilot interviews to modify the questions, interview process and language.

The semi-structured interviews were selected as a method to gain complementary information and to facilitate the adaptation of questions to each policy and case. The stakeholder interviews for each policy verified facts identified during the content analysis of policy documents and gaps in the information gathered in the cross-sector collaboration.

In the semi-structured interviews, the interviewees were asked to cast their minds back to the policymaking process time, review their files before the interview and then explain the policymaking process and the parties involved. The main issues in the interviews were stakeholders, structures, and processes of cross-sector cooperation in policymaking. The questions focused on the identification of stakeholders and sectors involved in policymaking groups and processes, and arguments used for HEPA policymaking and the possible benefits of, facilitators for or barriers to cross-sector cooperation.

The selected HEPA policy documents and interviews were mapped, coded and further analyzed with the help of the interview questions. Each HEPA policy and transcribed interview was reread, coded, sifted, charted and sorted according to the key issues and themes, confirming the stakeholders, the sectors involved and the benefits for cross-sector collaboration. The interviews were analyzed by the country-based research teams using an interpretative approach derived from a content analysis of the policy. The coding was done by the interviewers, and the accuracy of the content analysis was supported by independent assessments by the team members in the country research teams.

At the content analysis stage, the policy documents were reviewed by the REPOPA country teams so as to identify the stakeholders, processes and sectors included in cross-sector cooperation. When cross-sector cooperation issues were mentioned, the content was analysed to establish the way the citation supported the cross-sector cooperation or position in addressing the issue.

REPOPA developed an Ethics Road Map and Ethics Guidance Document to coordinate varying national ethics clearance procedures in the partner countries. Ethics clearance was done according to country-specific regulations and procedures [45]; however, irrespective of the country requirements, the informed consent of all participants was obtained.

## Results

The results section is broken into sections on stakeholder involvement, governance structures and coordination structures and processes. The section provides valuable understanding for the role of cross-sector cooperation in HEPA policy processes. An overview of the HEPA policies and related key institutions for the policies analyzed are presented in Table 1.

**Table 1:** Policies, key institutions and main topics of polices for HEPA policies in six European countries (see also [21])

Sector to introduce a policy document	Government level	Country	Policies studied and timeframe	Key institutions responsible for the policy documents
Regional authority	Regional	Denmark	The regional development strategy – Region of Zealand, 2012–15	Region of Zealand
Culture, sport and physical activity	Local	Denmark	The sports and physical activity policy of the Esbjerg municipality, 2011–12	Esbjerg Municipality
Health	Local	Denmark	Copenhagen City’s public health policy ‘Long Live Copenhagen’ 2011–14	City of Copenhagen
All sectors	Local	Denmark	The health policy of Odense municipality, 2011	Municipality of Odense
Health	National	Finland	Government resolution on development guidelines for health-enhancing physical activity and nutrition, 2008-2012	Ministry of Health and Social Affairs
Education and culture	National	Finland	Government resolution on policies promoting sport and physical activity, 2009	Ministry of Education and Culture
Transport and communication	National	Finland	National action plan for walking and cycling 2020, 2011-2020	Ministry of Transport and Communication
Regional authority	Regional	Finland	Regional strategy of health-related sport, Päijät-Häme 2008, 2009–20	Päijät-Häme Region: Regional sport and physical activity organization
Physical activity and sport	Local	Finland	Finland: City of Lahti health-enhancing physical activity strategy, 2007	City of Lahti; Department of physical activity, health enhancing physical activity, special physical activity
Health	National	Italy	National project for the promotion of physical activity based on the Ministry of Health policy ‘Gaining Health’, 2007	Ministry of Health Presidency of the Board of Ministers
Health	Regional	Italy	Emilia-Romagna regional prevention plan 2010–12	Regional Government Six Italian regions: Veneto, Emilia-Romagna, Piemonte, Lazio, Puglia, Marche
Regional authority	Regional	Italy	Tuscany Region, ‘Healthy roads’, 2010–12	Regional Government
Local administration	Local	Italy	‘Municipaliadi’ local policy for sport promotion for young people in Rome, 2011–12	City of Rome
Health and sports	National	The Netherlands	National policy ‘Health close People’, 2012–16	Ministry of Health, Welfare and Sports
Health and sports	National	The Netherlands	‘Sports & Physical Activity in the Neighbourhood’ 2012–16	Ministry of Health, Welfare and Sports
National and local authorities	National and local	The Netherlands	Youth on healthy weight (JOGG), 2010–15	National Consortium on Youth on a Healthy Weight, a combination of municipality and public–private organizations in the municipality
Youth and sport	National	Romania	National programme ‘Movement for Health’, 2003–ongoing	The Prime Minister of the Romanian Government Romanian Federation “Sport for All”
Youth and sport	National	Romania	National programme ‘Sport for All’ 3rd Millennium Romania – a Different Lifestyle 2001 – ongoing	Ministry of Youth and Sport (currently Ministry of Education, Research Youth and Sport – National Authority for Sport and Youth)
Education	Local	Romania	The protocol for organizing sport activities for children in Cluj County 2011-2012	Cluj County School Inspectorate Officials of the schools in Cluj-Napoca Cluj-Napoca Universities Consortium Cluj-Napoca City hall
Sports	National	England (United Kingdom)	‘Places People Play’, delivering a mass participation sporting legacy from the 2012 Olympic and Paralympic games	Minister for Sport and the Olympics British Olympic Association British Paralympic Association
Transport	Local	England (United Kingdom)	‘Herefordshire Council Local Transport Plan’ (2012–15) consultation document, 2012	Department of Health, Planning Department, Herefordshire Council

### Stakeholder involvement in HEPA policymaking

The main stakeholders mentioned in policy documents who were in charge of HEPA policies at the national level were different ministries ranging from health, sport, youth and culture to, for example, transport and communication. Based on interviews, some ministries had interdepartmental cooperation entities within the specific ministries.

Stakeholders identified from the policy documents and interviews were grouped into main partners and national, regional or local, non-governmental and public–private partners in policymaking (Table 1). The main partners often included central government, with ministries and institutional policymakers, national agencies, committees, advisory boards and educational institutes. Regional and local authorities, such as county or city councils, were driving forces in the regions and at the local level (in the municipalities). In addition, non-governmental organisations played an important role in HEPA policies and, in some countries, trade unions and employers’ organizations played a role in HEPA policymaking. Other partners in HEPA policymaking were enterprises (forming partnerships mainly at municipal levels on large projects and programmes), parishes and outspoken individuals. The national agencies often offered research evidence for policymaking, while committees, councils and advisory boards prepared the actual policy documents with a variety of approaches with influencing individuals, officers, professionals and participants.

Based on the content analysis of policy documents and interviews, multilevel approaches to HEPA policymaking were applied at national, regional and local levels; between public and private sectors; between international, national and local levels; between public sector, organizations, industries and commerce; and between media, business and non-governmental organisations. Cross-sector cooperation also occurred at many levels at the same time: cycle lanes were built by local authorities in cooperation with the private sector through funding from the national level, with education and/or transport sectors providing possibilities to enhance physical activity on cycle lanes through cycling to school or maintenance of cycling lanes by the transport sector.

According to the content analysis of HEPA policy documents, a genuine cross-sector approach was noticeable as many partners and cooperation entities were mentioned in national, regional and local level policy documents. However, the explicit definition of types of cooperation between sectors and modes of cooperation with an explanation of structures, processes and responsibilities in policy documents was rare. Based on the interviews, actual cooperation and co-working for policymaking was considered to be a challenge. Nevertheless, cross-sector cooperation included various types of intentions towards other sectors, such as education and training, human resources and training, young people’s associations and sport sector cooperation, as well as the involvement of non-governmental organizations, public sector organizations and the private sector.

Based on the interviews, the main partners of the HEPA policymaking often had the financing and managerial power to achieve particular policymaking objectives. Due to the objectives of policymaking and the fact that actual policy was achieved through the allocation of funding by the main institutions responsible, the process of policymaking had a formal institutional nature, and in some cases the policymaking process followed legal agreements or commonly used procedures, such as an accepted good practice process such as the so-called prevention cycle, as was the case in the Netherlands. According to the interviews, some policymaking processes for cross-sector cooperation also included dialogue and consultations with policymaking partners, such as in Denmark in the Esbjerg municipality, and in Italy. These open consultations sought to create guiding or facilitative frameworks, and often sought acceptance or responses for the planned activities, as in Odense in Denmark, in policies for various audiences.

### Governance structures of HEPA policymaking

The following section provides an overview of the coordination structures that have been set up in the six EU member states studied. Based on the content analysis of the HEPA policy documents, the governance and leadership of policymaking were in most cases held by the main partners at the national, regional and local levels, often in the form of an administrative entity of the state. At the national level, policymaking was governed by the responsible ministry and its working groups or committees. Based on the interviews, working groups or committees had subgroups or subcommittees with a variety of stakeholders as members. At the regional levels, interviewees confirmed that county councils or provincial governments nominated committees or working groups as the main leaders for policymaking. At the local level, interviewees explained that the municipal councils, politicians and stakeholders governed and drove the policymaking.

Based on the interviews, the policymaking processes included a complex set of interrelationships between financial resources, human resources and training, political will and intentions, and societal challenges with multiple factors and actors.

In Denmark, the policy process extended from local to regional levels, with multisectoral approaches internally in the municipal administration to gain cohesiveness, wealth and growth for the people. In addition, previous policies influenced new ones, which brought some continuity to the policymaking. Moreover, a participatory approach with hearings, partnerships and consultations with stakeholders and citizens was applied at the level of tokenism in policymaking. Other municipalities and examples from abroad also brought learnt lessons to HEPA policymaking in Denmark [46].

In Italy, based on the interviews, the policymaking process was characterized by multilevel policymaking and a policy trajectory, which meant that international policies influenced HEPA policymaking throughout ministries and downwards to local health units. The committed professionals were key stakeholders in policymaking in addition to providing political will and intentions. At the local level, a cross-sector approach between education, health and environment sectors characterized the HEPA policymaking.

According to the interviews, the Dutch policymaking for HEPA was led and regulated by the law on the prevention cycle and the length of the term of government. The prevention cycle refers to a regulated process of renewing policies based on public health data every fourth year. In this context, the policy process was influenced by politically-oriented values and the values and priorities of the leading political party as well as by the scientific evidence available. In local policymaking, municipalities created public–private partnerships across sectors and policymaking included substantial lessons learnt from abroad on a national level, but not generally on a local level. Both vertical and horizontal approaches of HEPA policymaking were used by local authorities for local solutions.

Romanian regional authorities organized hearings and field visits to make policy for sport activities for children according to the interviews done in Romania. The policymaking considered epidemiological data, political motives, intentions and will in addition to requirements set by laws for policy, such as the ‘sports for all’ principle. The Romanian national policy for HEPA was an administrative policymaking process to guide local authorities in their activities. Other characteristics of the Romanian national policy for HEPA were references made to international policies, continuity of policies and formal decision-making routes.

In the United Kingdom, the HEPA policies considered for this analysis can be characterised as being part of nation building through engaging people in physical activity prior to the 2012 Olympic Games and as a policy contracted to non-governmental organisations for the promotion of statement-type policy. In the local HEPA-related policy for transport, the designated bodies prepared the plan based on administrative requirements.

Finnish policymaking can be characterized by working groups with invited representatives from various stakeholders. However, the role and methods of work or how issues were selected for inclusion in the policy were mostly dependent on the participants rather than those arising from research evidence or evidently from politicians. The nomination of members of the working groups was meant to provide representativeness of a wider range of interested stakeholders or organizations. However, wider stakeholder hearings and consultations were organized with a modest approach in only one of five policymaking processes.

Table 2 presents the key coordination and cooperation structures and processes for HEPA policies and institutions of each country for cross-sector cooperation in HEPA policies (see also [35]). Some countries may have several governance structures for one policy. Ministerial, regional or local administration linkages meant a variety of structures and processes. Those structures included the whole of government or horizontal management approaches to tackle social, economic or environmental challenges. The collaboration was often across government within a level or between levels. Cross-sectoral committees were referred to as being evidence-based and supporting the topics, such as for the collection of information, commissioning or performing research, engaging in public debates, informing ministers, or aggregating information for policymaking processes. National, regional and local politically elected groups included city councils, regional councils or parliamentary groups for HEPA-related issues.

**Table 2:** Key coordination and cooperation structures and processes for HEPA policies in six European countries

Country/Coordination and cooperation structures	Denmark	Finland	Italy	The Netherlands	Romania	England (United Kingdom)
Government/Regional/Local committees or working groups with cross-sector representatives	X	X	X	X	X	X
National/regional/local politically elected councils	X	X	X			
Contacts between public sector officers responsible for HEPA between levels		X	X	X	X	
Established system of policymaking process				X		
Intersectoral committees or working groups for HEPA	X	X				
Scientific advisory groups/institutes/individuals		X			X	X
Steering committees	X			X		
Administrative working groups including only public sector officers	X					
Private sector involvement in policymaking	X		X	X		
Formal consultation on HEPA policy for stakeholders	X	X		X	X	X
Public hearings for citizens	X	X	X			
Field visits to make a policy					X	

### Key coordination structures and processes

According to the interviewees, the cross-sector cooperation in Italy, Denmark and the Netherlands was an evidence-informed choice and an approach for HEPA policy to increase its effectiveness. In the Netherlands, the interviewees claimed that cross-sector cooperation depended on responsibilities in each unit and its subject area in the administration. Sometimes, working across sectors made it unclear who was accountable for specific changes. Every department, ministry or institute had a need to show its value and impact in cross-sector cooperation. In addition, cross-sector cooperation work was dependent on the persons involved, especially in Italy. The interviews in the Netherlands revealed that stimulation of cross-sector cooperation also depended on support from the highest political level. Sometimes, the quite specific views of political leaders, the role of research institutes or interest groups, and some themes were kept within ministries in the Netherlands. Cross-sector cooperation was not easy in developing the Health Close by People policy in the Netherlands due to competition on funding between different national institutes and the prescriptions in the law on the prevention cycle as a regulated policy process. A similar competitive edge and schism was reported in interviews in Finland between sports and health sectors, due to their sector-specific interests and pride in defending their own funding and services. Based on the interviews in the Netherlands, cross-sector cooperation was used to tackle obesity, since the common aspiration was that local integrated approaches worked best. The integrated approach meant that it focused on lifestyle, but also on healthy, physical and social surroundings. The idea was that all the factors that may influence the situation where a person lives should be considered.

Based on the interviews, the inclusion of various aspects of citizens’ perspectives on cross-sector cooperation was considered justifiable, and was often based on joint planning and evaluation of policies. Such inclusion was considered a value in Denmark and Italy. Cross-sector cooperation was mentioned in national as well as local policies as a model for joint planning in Romanian sport, education and health sectors within the Movement for Health policy document. According to the policy documents, the county council, county school inspectorate and county sport and youth department were partners in joint planning of the local policy entitled “The protocol for organizing sport activities for children in Cluj County”. Based on the interviews in Romania, in the actual implementation, at least of local policy, only one of the partners was actually involved (in this case Cluj County Council). Although cross-sector cooperation was mentioned in the policy documents analysed, it was usually not implemented in the real policy context. In Romania, the cross-sector cooperation was mentioned in policies, but was mentioned in the interviews as being more relevant in the implementation phases of the policies.

For the governance of cross-sector cooperation, HEPA policies described processes as strategic choices for society to work together for equality and the preconditions for HEPA, but also to clarify the processes and division of work for implementation (Finland, Denmark). According to the interviews, financial frameworks played an important role in cross-sector cooperation to pool resources and ensure sustainability of actions and an appropriate use of resources (Finland, Denmark and United Kingdom). Based on the interviews, cross-sector cooperation also broadened and ensured competencies and human resources for HEPA policies (Finland, Denmark, Italy and Romania). The interviewees confirmed that the values of society (communality or public health as a shared value and responsibility) also determined the cooperation, though they also intensified the interests and values of HEPA policies among other sectors to increase cross-sector cooperation (Finland, Denmark). The interviewees mentioned that cross-sector cooperation was also justified by multi-disciplinary HEPA and by the health-in-all-policies (HiAP) perspectives (Italy, Denmark). Moreover, the public sector reforms mandated cooperation with other sectors and closer links between various sectors (Denmark) according to the interviewees. A summary of the overall coordination features is given in the Table 3.

**Table 3:** Overall coordination features for HEPA policies in six European countries

Country	Overall coordination features
Denmark	Regional/local responsibilities; decentralized; citizens hearings
Finland	Rather centralized at all levels; ministerial responsibility; network arrangements
Italy	Rather decentralised; network arrangements; strong individuals; private sector involvement
The Netherlands	Rather centralized at strategic level with local network arrangements including private sector; strong central agencies; law-based approaches for policymaking
Romania	Centralized responsibility with fragmented network arrangements; emphasis on local circumstances
England (United Kingdom)	Rather centralized at strategic levels; ministerial responsibility with network arrangements at national and local levels

## Discussion

The main focus of this paper was to find insights into a formal structure of cross-sector cooperation in HEPA policymaking in six countries, identifying the actual cross-sector cooperation mechanisms, their role in physical activity, health and other sectors, and the perceived picture on cross-sector cooperation. The contribution of this paper to knowledge about cross-sector cooperation in HEPA in unique. Other published papers focus mostly on the content of HEPA policies [7–11], the content of recommendations for physical activity [5] or how to prepare successful policies [6]. However, Rütten et al. [47] considered that physical inactivity as a policy issue should be addressed through a broad scope of activities and needing cross-sector cooperation between various levels. The structural variation displayed between countries (encompassing variation in centralisation versus decentralisation), lead agencies and networks is large, and their impacts on coordination ‘quality’ were complex and ambiguous to appraise. Efforts to enhance cross-sector cooperation with a structured and step-by-step approach for policy changing processes could improve success [48]. One solution does not fit all cases, but the development of coordination structures, which increases harmonisation and coherence of actions between the stakeholders, would make choices acceptable and lead to them being coherently promoted for healthy life (World Economic Forum [49]). Promotion of HEPA and the prevention of sedentarism are interdependent on action in other sectors, while the diversity of choices, means and methods puts pressure on multi-dimensional coordination. Finding solutions and balancing the coordination within and between national, regional and local governments with civil society and the private sector are complicated and specific to their contexts, but are worth trying out for better health and well-being [47, 48, 50].

The linear model of knowledge transfer has been challenged [41], and the involvement of stakeholders in framing problems has also been recognized [48, 51–53]. In addition to the need to increase more participative approaches with collaboration, the production of knowledge for policymaking includes attributes such as the interdisciplinary approach, links between theory and practice, participatory approaches employing different knowledge forms and including a reflection on the variety of knowledge elements [54]. It is generally accepted that there are different knowledge forms with different powers and cultural significance. In a policymaking setting, existing power structures and inequalities between stakeholders create opportunities for mutual learning and communications in policymaking, when the separate efforts of different sectors have failed and potential failures cannot be fixed by one sector only. This can lead to improved practices in cross-sector cooperation and interventions for health [55].

The data sources employed (policy analysis of documents and interviews) provided a rich empirical background against which arguments were assessed. However, the study gained a small, though important, glimpse of the comprehensive alternatives of HEPA organizations involved in this policy area. Therefore, a fairly modest number of observations implied that conclusions must be drawn with caution. Nevertheless, the data suffices to illuminate how various organisations have organised their policymaking practices, governance structures, responsible institutions for recent HEPA policy documents and key coordination and cooperation structures for HEPA policies.

The main methods of the cross-sector policy process can be divided into stakeholder involvement, governance structures and coordination structures and processes. In stakeholder involvement, citizens’ hearings included activities such as dialogues with various population groups, councils, committees and organizations and organized events for policy development and information dissemination. Governance structures included policy and politics discussions, comments on writing, seminars and conferences between politicians, committee members, working groups, steering committees, advisory groups and stakeholders on local, regional and national levels. Coordination structures and processes included administrative processes, such as training, writing and information dissemination for officers and others concerned, as well as processes associated with the acceptance of policies. Similar knowledge translation tools have been reported by several researchers [56–60]. Flitcroft et al. [61] concluded that embedding public review of the evidence for policymaking may be more successful in delivering evidence-informed policy than the voluntary methods often used.

Various stakeholders participated in the cross-sector cooperation within HEPA policymaking processes. The stakeholder involvement varied from ad hoc processes to a law-based approach to cross-sector cooperation. Legal mandates may over-prescribe process at the expenses of flexibility. Agencies may comply with partnering in reports without engaging in good-faith collaboration. Also, enforcement by law might be difficult [62]. The key stakeholders responsible for the HEPA policymaking were the national ministries, county councils and city councils in all countries, as these entities also had the power and financial resources to steer the implementation phase of the policies. The means, methods and forms of participation were defined mainly by the ministries rather than by other stakeholders. There were also various forms of participatory approaches, from individuals to citizens’ hearings. However, HEPA policymaking involved experts from various stakeholder institutions. Furthermore, committees for policymaking were nominated so that they represented organisations and therefore were considered as presenting citizens’ voices in policymaking. However, organizations advocated on the issues of their members, not necessarily the issues of the whole population concerned.

By defining cooperation sectors, modes of cooperation and governance structures in policymaking phases, intensified cooperation can occur between sectors, topics and policymaking processes. Governance and collaborative processes can be strengthened by engaging stakeholders in policymaking. The considerable advantages of involving stakeholders in policymaking may improve understanding, delivery of the policy output and support for the policy implementation [63]. One instrumental benefit of participation is the possibility to establish a collaborative relationship and a degree of conflict resolution. Stakeholders often appreciate the advantage of hearing different perspectives and gaining better understanding of others’ views. There is also a recognized difficulty in obtaining inputs from all stakeholders and therefore, in policymaking, the awareness of skewed representation and process failures impedes the goal of fair and effective participation. Similarly, the Health in All Policies (HiAP) approach is one that promises interventions, as it systematically addresses health in policymaking by targeting broad health determinants. In HEPA, there are various social mechanisms and contextual factors that characterize successful practices in implementing HiAP in various countries. Therefore, the HiAP approach can support cross-sector cooperation [64–66].

The main coordination structures and processes for cross-sector cooperation were cross-sector working groups and committees, inter-sector contact between various stakeholders, scientific advisory groups and in some cases the hearings with various stakeholder groups. However, many other options remain for cross-sector cooperation. Laegreid et al. [63] mentioned novel coordination practices such one-stop shops; policy networks; new/restructured agencies and ministries; common shared strategies, programmes or objectives; systems for exchanges of information; joint planning/working groups (temporary, long term, permanent); specific joint entities (advisory, executive or regulatory); special appointments with coordination responsibilities, i.e. high-level leaders and advisers; interagency collaboration units; strategic units and reviews; intergovernmental councils, teams, task forces; a lead agency force; cross-sectoral policy programmes; digital-era governance solutions; and specific budgetary tools to encourage achieving common goals [63]. From all these coordination structures, the closest to HEPA cross-sector coordination were policy networks, joint planning/working groups and cross-sectoral policies. Therefore, ample space remains to further develop the cross-sector cooperation in HEPA policymaking and contribute to the development of methods, means and processes for cross-sector cooperation.

The critical questions for cross-sector cooperation are how to develop and share practical knowledge in cross-sector cooperation and how different sectors are equipped for it. Research evidence can be used either for ‘enlightenment’ or ‘ammunition’ in cross-sector cooperation, because decisions are also based on ordinary knowledge and social interaction [67]. Concentrated interests play an important role, as most public health policies are formulated, adopted, and amended over time within a relatively small network of elected officials, legislative and administrative staff, interest group leaders, researchers and reporters, whose knowledge and activities are devoted principally to a specialised policy area [35]. Policymakers and professionals play an important role in nearly every aspect of physical activity policy such as lobbying administrative agencies, stimulating grassroots activity, developing cross-sector cooperation and cooperative planning [48].

Cross-sector cooperation is participation with the co-production of goals and resource-sharing between stakeholders, which may provide a mechanism for collaborative decision-making [12]. In many cases, state support is preferred for collaborative groups with differing stakeholder priorities in policymaking. Clear responsibilities, goals, communication, learning and adaptation of HEPA policies may be needed to improve the success of cross-sector cooperation in HEPA policymaking [47]. Specific resources available for cross-sector cooperation would facilitate empowerment and enhance the management and outcomes of cross-sector cooperation when health in all policies is applied [50].

## Conclusions

International and national policies urge us towards cooperation between sectors so as to solve complex problems. This study showed that approaches to cross-sector cooperation differ in terms of substantive issues and sectors, but also in relation to the level and forms of governance.

Successful cross-sector cooperation benefits form the content analysis of HEPA policy documents and stakeholder interviews, joint planning and evaluation, financial frameworks, agreed methods of work, communication lines and valued processes of cross-sector cooperation [50]. In this study, the HEPA policymaking processes involved various stakeholders, from politicians to administrative officers, scientists, outspoken individuals, various types of organizations, consultants and citizens.

Competing institutional interests to some extent acted as constraints on cross-sector cooperation within policymaking. Other general institutional tensions identified included the tension between ministerial hierarchical steering on the one hand and hesitation in using participative forms of policymaking on the other, which partly reflected the feeling of undermining the role of expert knowledge and electoral legitimacy. Complex problems such as the promotion of HEPA require cross-sector cooperation with open discussions. These constraints, in addition to an unclear definition of HEPA in national policy contexts, create tensions and obstacles for cross-sector cooperation and stakeholder involvement. Lack of cross-sector cooperation is also dependent on gaining voice, legitimacy and presence as part of the governance of HEPA policies. Cross-sector cooperation should be an aim in itself in HEPA policies to tackle present health challenges, but also to demand measures, hearings and reports on how cross-sector cooperation should respond to the complex issues of society.
